# Prognosis Prediction of Uveal Melanoma After Plaque Brachytherapy Based on Ultrasound With Machine Learning

**DOI:** 10.3389/fmed.2021.777142

**Published:** 2022-01-21

**Authors:** Jingting Luo, Yuning Chen, Yuhang Yang, Kai Zhang, Yueming Liu, Hanqing Zhao, Li Dong, Jie Xu, Yang Li, Wenbin Wei

**Affiliations:** ^1^Beijing Tongren Eye Center, Beijing key Laboratory of Intraocular Tumor Diagnosis and Treatment, Beijing Ophthalmology & Visual Sciences Key Lab, Medical Artificial Intelligence Research and Verification Key Laboratory of the Ministry of Industry and Information Technology, Beijing Tongren Hospital, Capital Medical University, Beijing, China; ^2^InferVision Healthcare Science and Technology Limited Company, Shanghai, China

**Keywords:** uveal melanoma, machine learning, B-scan ultrasonography, follow-up, plaque brachytherapy

## Abstract

**Introduction:**

Uveal melanoma (UM) is the most common intraocular malignancy in adults. Plaque brachytherapy remains the dominant eyeball-conserving therapy for UM. Tumor regression in UM after plaque brachytherapy has been reported as a valuable prognostic factor. The present study aimed to develop an accurate machine-learning model to predict the 4-year risk of metastasis and death in UM based on ocular ultrasound data.

**Material and Methods:**

A total of 454 patients with UM were enrolled in this retrospective, single-center study. All patients were followed up for at least 4 years after plaque brachytherapy and underwent ophthalmologic evaluations before the therapy. B-scan ultrasonography was used to measure the basal diameters and thickness of tumors preoperatively and postoperatively. Random Forest (RF) algorithm was used to construct two prediction models: whether a patient will survive for more than 4 years and whether the tumor will develop metastasis within 4 years after treatment.

**Results:**

Our predictive model achieved an area under the receiver operating characteristic curve (AUC) of 0.708 for predicting death using only a one-time follow-up record. Including the data from two additional follow-ups increased the AUC of the model to 0.883. We attained AUCs of 0.730 and 0.846 with data from one and three-time follow-up, respectively, for predicting metastasis. The model found that the amount of postoperative follow-up data significantly improved death and metastasis prediction accuracy. Furthermore, we divided tumor treatment response into four patterns. The D(decrease)/S(stable) patterns are associated with a significantly better prognosis than the I(increase)/O(other) patterns.

**Conclusions:**

The present study developed an RF model to predict the risk of metastasis and death from UM within 4 years based on ultrasound follow-up records following plaque brachytherapy. We intend to further validate our model in prospective datasets, enabling us to implement timely and efficient treatments.

## Introduction

Uveal melanoma (UM) is the most common aggressive ocular tumor in adults. The annual incidence rate per million people is 6 in non-Hispanic whites (1) and 0.3–0.6 in Asians (2–4). Although new techniques such as proton beam therapy have been introduced (5), plaque brachytherapy, mainly using iodine-125, remains the dominant option as an eyeball-conserving treatment for UM. In the United States, the ratio of plaque brachytherapy is increasing each year, which accounts for more than 50% recently (6, 7). The same case was also seen in our eye center. However, patients with UM have high mortality with approximately 50% of patients developing metastatic disease and eventually dying within 5 years (8, 9). Therefore, it is important to predict the metastasis risk and long-time survival accurately.

Several factors have been proven to correlate with patient outcomes. These include tumor size and location, as well as related features such as retinal detachment, extrascleral extension, and retinal invasion (10, 11). The most significant factor for melanoma-specific mortality prediction is dependent on tumor-specific genetic alterations and histopathologic factors including epithelioid cell type, monosomy 3 and 6p gain, and loss of BAP-1 gene (12). Gene expression profiling (GEP) of 15 genes was divided into class 1 and class 2 UM, those with the class 2 GEP have a greater rate of metastasis and mortality compared to class 1 GEP. However, fine-needle aspiration is not available in most cases for patients with UM treated by plaque brachytherapy. Therefore, we wish to construct a prediction model with more readily accessible clinical data.

Ultrasonography, a cost, and time-effective non-invasive examination is the most used application for determining the dimensions of a posterior UM. And it is essential throughout follow-up for tumor measurement (13). Tumor regression has commonly been evaluated as a percentage change from initial tumor thickness measured with B-scan ultrasonography. According to the Collaborative Ocular Melanoma Study, a 15% increase in tumor thickness after brachytherapy should be considered as a failure. Many previous studies have shown that such local treatment failure (14–17) and rapid regression of tumors after plaque brachytherapy (18, 19) predict a lousy prognosis.

**Figure 1:**
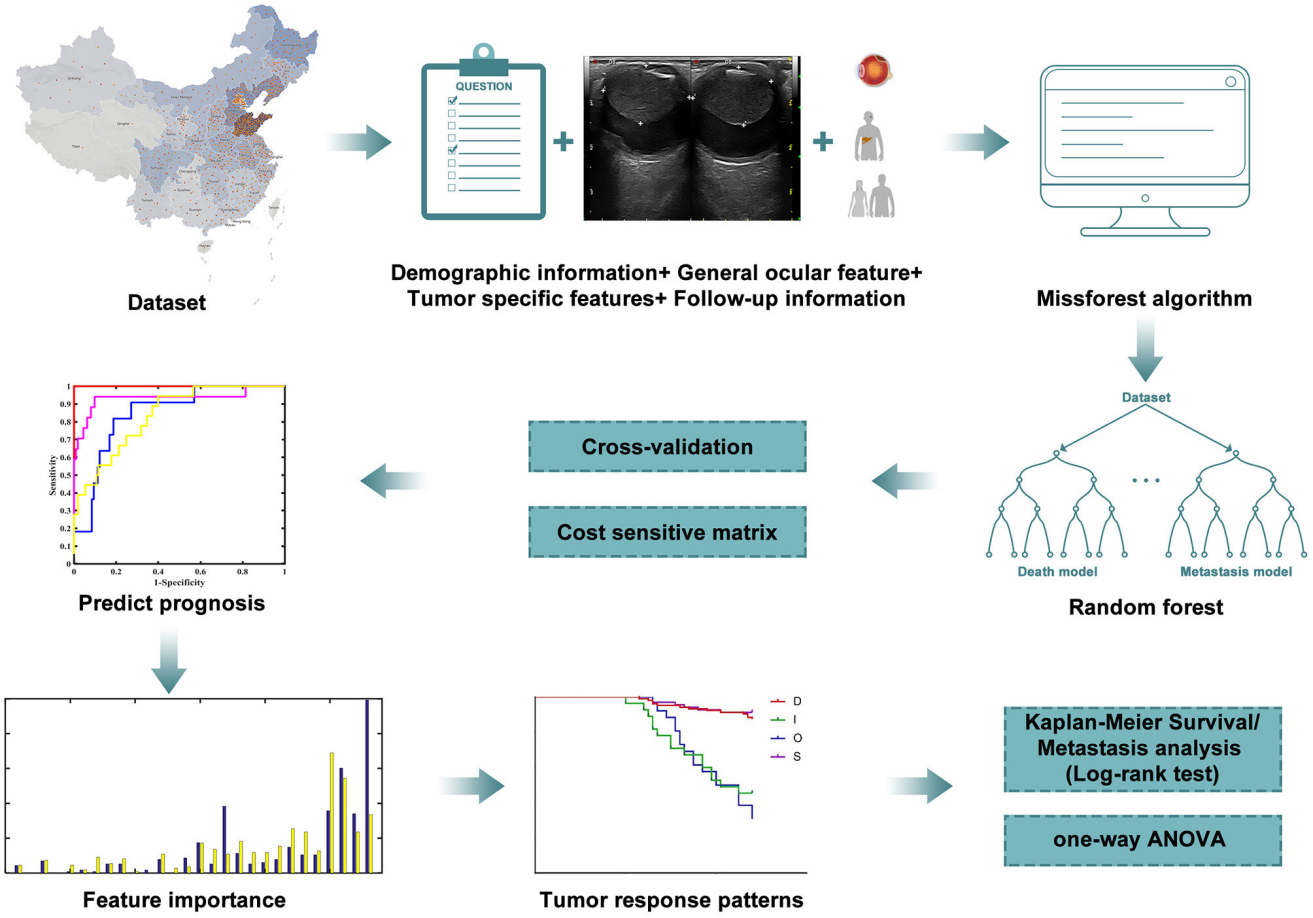
Workflow of the research. Machine learning was carried out according to the clinical and follow-up data from patients to predict the prognosis. Results of metastasis and survival analysis are based on filtered data.

Previous models based on clinical and demographic characteristics have been developed to predict individual patient prognosis after UM treatment (20–26). To our knowledge, this is the first report that describes a mathematical model for patients with UM after iodine-125 plaque brachytherapy using postoperative follow-up ultrasound data. The present study investigates the prognostic value of dynamic morphometric parameters to predict 4-year survival and metastasis status (Figure 1).

## Materials and Methods

### Source of Data

This is a retrospective, single-center study conducted in the Beijing Tongren Eye Center. The study population included adult patients that were clinically diagnosed with UM from July 2007 to December 2016. Generally, iodine-125 plaque brachytherapy was used for tumors with a thickness of <10 mm in our center. The standard dose of irradiation was 100 Gy to the apex of the tumor. However, patients who were refractory to other treatments and strongly requested it were also treated by brachytherapy.

### Selection of Participants

**Figure 2:**
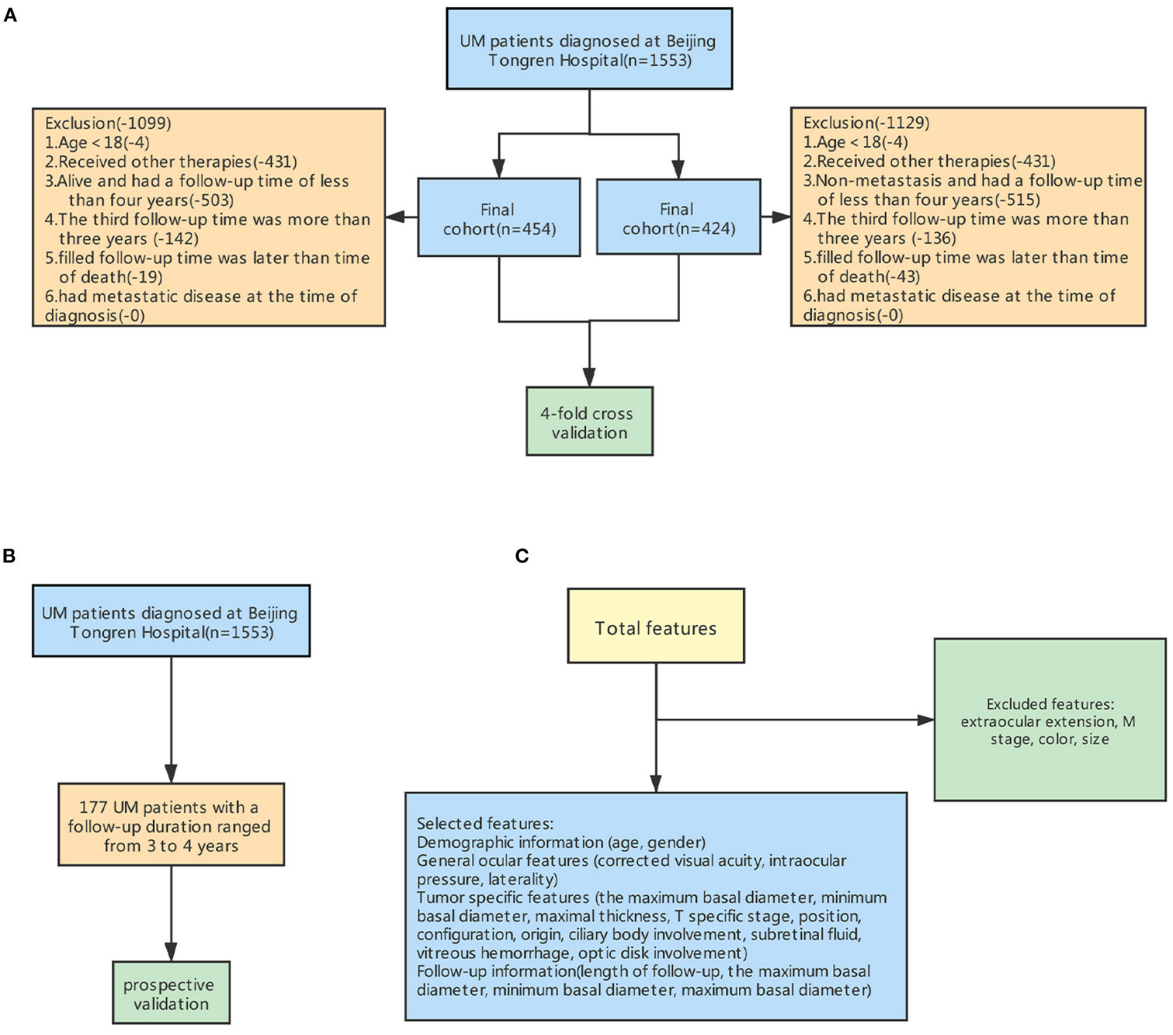
Steps for excluding features and samples to preprocess the input dataset for machine learning. **(A)** The workflow for excluding some samples. **(B)** Prospective validation **(C)** The workflow for excluding some features.

Patients who were diagnosed with UM at the Beijing Tongren Eye Center and subsequently received brachytherapy were included in this study. The exclusion criteria were: (1) age <18 years, (2) received other therapies, (3) alive and had a follow-up time of fewer than 4 years, (4) the third follow-up time was more than 3 years, (5) filled follow-up time was later than the time of outcome, (6) had metastatic disease at the time of diagnosis. Finally, 454 patients were included to construct the model for predicting death and 424 patients to build the model for predicting metastasis (Figure 2A). Moreover, 177 surviving patients with UM had a follow-up duration ranging from 3 to 4 years. They will be included in the prospective validation of our models in future studies (Figure 2B).

### Data Collected

The age, gender, and involved eye were recorded from each patient's record during the initial interview. The presence of subretinal fluid, optic disk involvement, vitreous hemorrhage, ciliary body involvement, tumor thickness, minimum and maximum tumor diameter, tumor shape and position, intraocular pressure and visual acuity, photographs, and ultrasound records were collected from the preoperative medical records. Several strategies, including fundus photography, fluorescein angiography, indocyanine green angiography, standardized echography, and orbital MRI, were conducted to assist diagnosis. Tumors were staged according to the American Joint Committee on Cancer (AJCC) consensus. We excluded duplicate factors and factors that did not differ among groups (Figure 2C).

Ultrasound images were reviewed by two independent radiologists with at least 5 years of experience in interpreting ocular ultrasound images. The radiologists were blinded to the clinical data. When two radiologists failed to reach a consensus through their independent assessment, the image would be reviewed jointly to ultimately achieve agreement. They measured the tumor's thickness from the inner surface of the sclera to the tumor apex and maximum basal diameter. Thickness and the minimum basal diameter were measured from two meridians, along with the maximum basal diameter and perpendicular to it. Representative digitized scans were stored at the time of each diagnostic and follow-up visit.

### Missing Value Completion

There were some missing data values due to the loss of clinical data and some missing features. The missforest algorithm (R package missForest) was used to fill in the blank values in the dataset (27). Missforest iteratively filled all features with missing values by predicting missing values from existing values. The order for filling missing values was from features with the fewest missing value to the feature with the most missing values. Moreover, numerical features and nominal features were predicted with Random Forest (RF) regression and classification, respectively. The follow-up information of patients with less than three visits was also filled, and the length of follow-up was less than that of outcome events.

### Prediction Model

Machine learning is a powerful tool for mining the hidden relationships in our dataset which included imaging (28–32), genetic (33), clinical (27, 34), multi-modal sensor data (35–37), and other sources (38). RF is a type of ensemble learning method which encapsulates multiple decision trees to vote the classification results. The decision tree is a basic machine learning method that applies tree data structure to recursively split the whole dataset into multiple subsets. Finally, the samples in each leaf node either belong to one class or own more features could be used to be split, namely, the class of each sample can be inferred according to the paths from the root node to leaf nodes in the tree (39–42). In our research, the RF model was used to construct models of whether a patient will survive for more than 4 years and whether the tumor will metastasis within 4 years after plaque brachytherapy. This was done using demographic attributes, clinical features, and follow-up records.

Additionally, all datasets used were imbalanced. Therefore, the most convenient, cost-sensitive method (43) was used to tackle this problem and assist RF in constructing the models. Synthetic Minority Oversampling Technique (SMOTE), the simplest oversampling algorithm, is typically used to enrich the minority in each training set of the internal cohort. Numerical and nominal features are preprocessed differently in terms of measuring the distance of two samples. However, we did not adopt this method because we cannot guarantee the ratio for generating more minority class samples. It will also import some noise into the dataset. The numerical and nominal features were separately oversampled and then merged. The under-sampling method (44) randomly deleted some majority samples in the training set, which was not suitable for our study because the follow-up dataset is precious. We cannot sacrifice the majority class to trade off the minority class. Similar to the multi-objective optimization, the cost-sensitive method (43) leveraged another objective function (cost function) and accuracy function in constructing a machine learning model. The number of trees in RF was primarily set as 500 when experiments were carried out. Four-fold stratified cross-validation was used to evaluate the performance of RF fairly, and the subjects in each fold were independent (a patient owns only one entry of data).

### Statistical Analysis

The baseline characteristics of enrolled participants were presented and compared between survivors and non-survivors by applying either Student's *t*-test, Chi-square test, and Mann-Whitney U-test as appropriate. Continuous variables were characterized as mean (standardized differences [SD]) or median (interquartile range [IQR]), while categorical or ranked data were reported as count and proportion. One-way ANOVA and Kaplan-Meier analysis were used to evaluate tumor regression patterns. All calculations were performed in Statistical Package for the Social Sciences (SPSS) version 26 and GraphPad Prism version 7. Random forest was performed using Python 3.7.3 (Wilmington, DE, United States) and MATLAB R2016a. Accuracy, sensitivity, specificity (32, 45, 46), Receiver Operating Characteristic (ROC) curve, Precision-Recall (PR) curve, and Area under Receiver Operating Characteristic Curve (AUROC) were used to evaluate the performance of models.

## Results

### Baseline Characteristics

**Figure 3:**
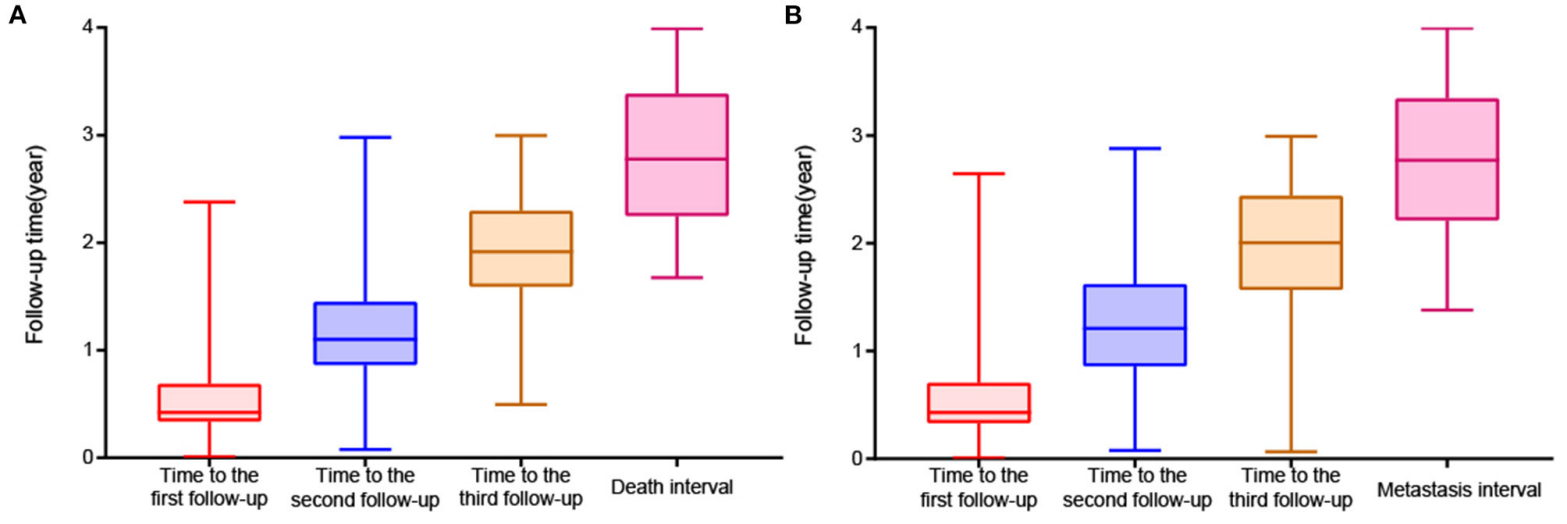
Boxplot of follow-up time. **(A)** Death analysis. **(B)** Metastasis analysis.

A total of 454 patients with UM treated by plaque brachytherapy were included in the death analysis. 210 (46.3%) were male. UM occurred in 248 right eyes and 206 left eyes. The mean age was 46.3 ± 11.7 years. We used the criteria of the AJCC consensus to determine that there were 41 T1 stage tumors, 213 T2 stage tumors, 137 T3 stage tumors, and 11 T4 stage tumors. In total, 48 of 424 (11.3%) patients developed metastasis during the follow-up period, and 52 of 454 (11.5%) patients died. The baseline characteristics are compared in Supplementary Table 1. The stage, position, ciliary body involvement, subretinal fluid, maximum and minimum basal diameter, and follow-up data were significantly correlated with death and metastasis. The median period was 154 [IQR:124, 251], 402 [IQR:315, 530], 700 [IQR:581, 839] days respectively for the three follow-up visits (Figure 3).

### Evaluation of Model Performance

**Figure 4:**
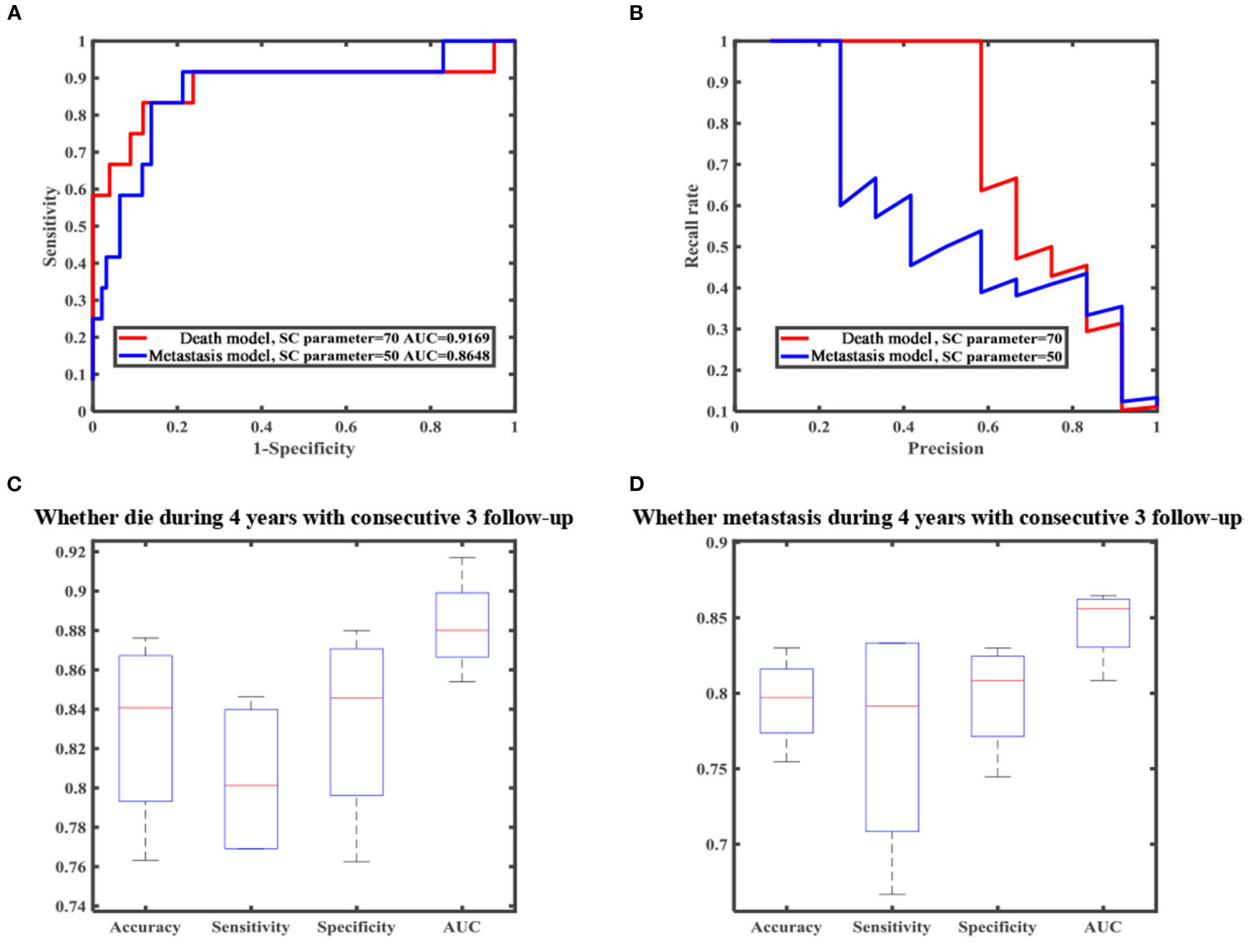
Machine learning result of death and metastasis model. **(A)** Receiver operating characteristic (ROC) curve. **(B)** Precision-recall (PR) curve. **(C)** Boxplot of all metrics for predicting death. **(D)** Boxplot of all metrics for predicting metastasis.

**Figure 5:**
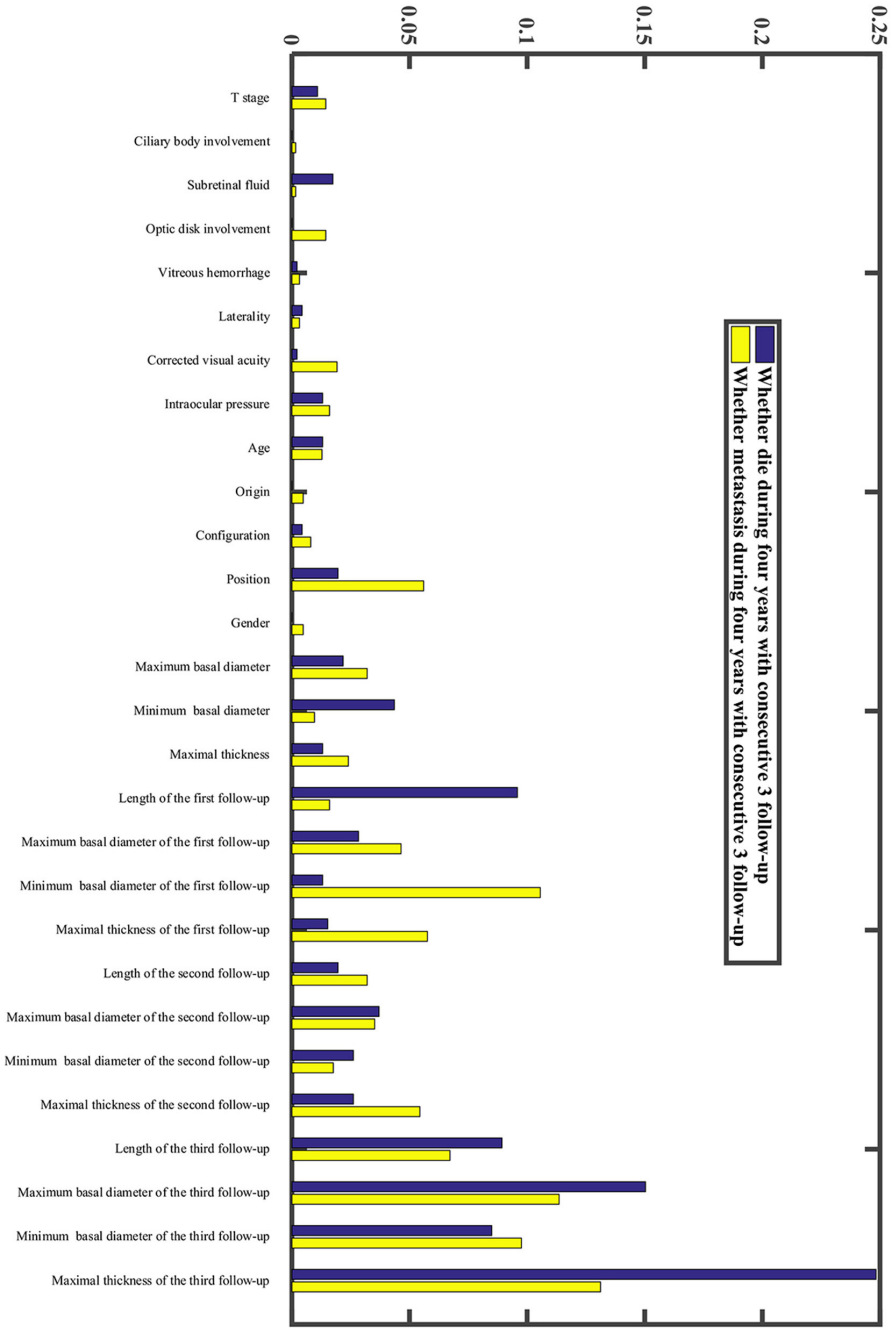
Relative importance of each factor in the machine learning model.

In our research, we developed a model to predict death 4 years after treatment, (Figures 4A,B) with 70.51% sensitivity, 56.96% specificity, and overall diagnostic accuracy of 58.51% using the first follow-up data. The overall performance of the prediction model was improved when three follow-up records were included. The performance was raised to a sensitivity of 80.45%, a specificity of 83.35%, and overall diagnostic accuracy of 83.02% (Figure 4C, Supplementary Table 2). Due to imbalanced datasets, we used a relatively high cost-sensitive parameter to increase sensitivity. A higher sensitivity means that patients with poor prognoses are more likely to be detected in clinical practice and radical treatments can be undertaken earlier to improve patient outcomes. The maximum basal diameter was the top-ranked preoperative factor related to death within 4 years after surgery (Figure 5). Position, preoperative minimum basal diameter, corrected visual acuity, and intraocular pressure was also clearly correlated with death. In addition, the span of records from the follow-up was remarkably correlated with predicting death. Thus, obtaining data from three follow-ups had the greatest impact on accuracy.

Moreover, we constructed a model to predict four-year metastasis status (Figures 4A,B), with 66.67% sensitivity, 69.42% specificity, and 69.10% accuracy. We then incorporated additional follow-up information to achieve a sensitivity of 77.08%, a specificity of 79.79%, and overall diagnostic accuracy of 79.48% (Figure 4D, Supplementary Table 2). The model for predicting death did perform better than the one for metastasis. We found that the maximum basal diameter, intraocular pressure, minimum basal diameter were the most critical factors (Figure 5). Similarly, additional follow-up information beyond the first collection was significantly related to successfully predicting metastasis. Tumor thickness recorded in the third follow-up was the most important information.

### Regression Pattern

**Figure 6:**
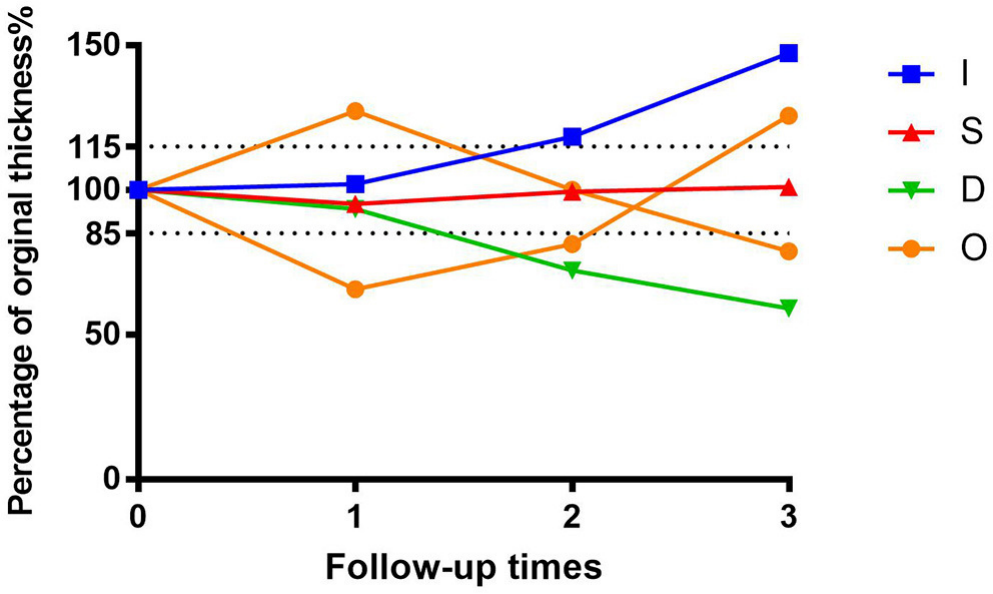
Percentage graph of initial tumor thickness vs. duration of follow-up after iodine 125 brachytherapy for patients with uveal melanoma by tumor regression patterns (D vs. S vs. I vs. O).

**Figure 7:**
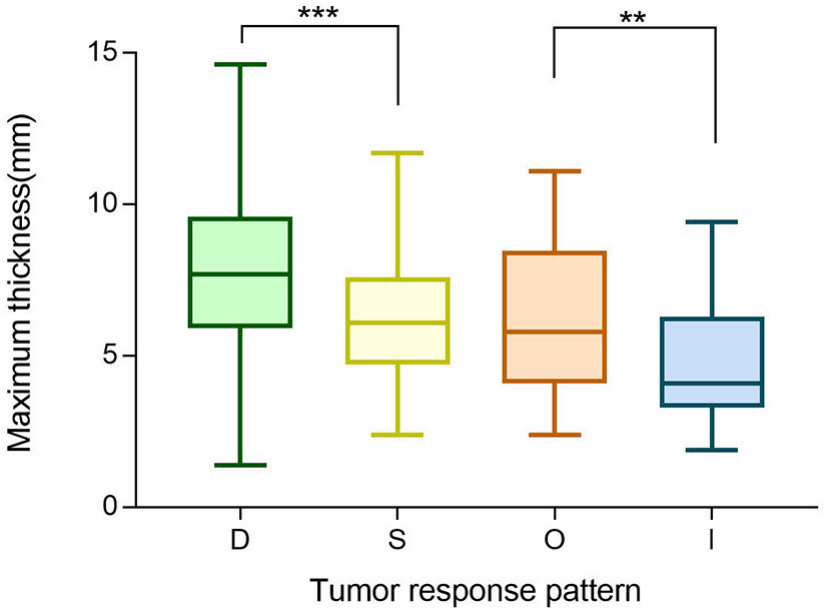
Boxplot of preoperative maximum thickness of different tumor regression patterns (D vs. S vs. O vs. I). **P ≤ 0.05, ***P < 0.001.

We next investigated investigate the importance of tumor thickness after treatment. We classified the tumor response to brachytherapy into the following four main patterns (47, 48) (Figure 6). Pattern D (decrease) involved at least one follow-up visit, the thickness decreased by at least 15% compared to the preoperative period, and two other visits also showed a decrease in thickness. Pattern S (stable) indicates there was < a 15% change in thickness. Pattern I (increase) is defined by at least one follow-up visit, the thickness increased by at least 15% compared to the preoperative period, and thickness also increased at two other visits. Pattern O (others) indicates an irregular change in thickness. Preoperative tumor sizes of different patterns are listed in Table 1. It was found that the tumor regression rate increased with increasing tumor thickness (*P* < 0.001) (Figure 7).

**Table 1:** Preoperative tumor size of different patterns.

	**D**	**S**	**I**	**O**	***P-*value**
Maximum basal diameter, mm	11.7 ± 2.8	12.0 ± 2.5	12.1 ± 3.6	12.5 ± 3.7	0.311
Minimum basal diameter, mm	10.4 ± 2.7	10.5 ± 2.4	10.6 ± 3.2	11.1 ± 2.7	0.409
Maximum thickness, mm	7.6 ± 2.3	6.3 ± 2.0	4.9 ± 2.0	6.2 ± 2.4	<0.001

**Figure 8:**
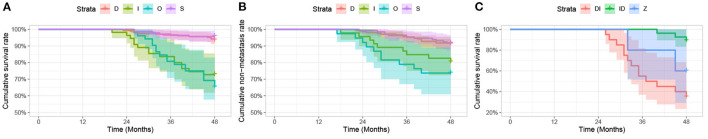
Kaplan-Meier analysis. **(A,B)** Kaplan-Meier analysis of survival curve **(A)** and non-metastasis curve **(B)** for patients with uveal melanoma after iodine 125 plaque brachytherapy by tumor regression patterns (D vs. S vs. I vs. O), expressed in three follow-up visits. **(C)** Kaplan-Meier analysis of survival curve for patients with uveal melanoma after iodine 125 plaque radiotherapy by tumor regression patterns (DI vs. ID vs. Z), expressed in three follow-up visits.

As shown in Figures 8A,B, there is a statistical significance relating metastasis and death (*P* < 0.001) to different tumor regression patterns. Patterns D/S were associated with a significantly better prognosis than the I/O group. Then, we further categorized the O group into three subtypes: DI (decrease followed by increase), ID (increase followed by decrease), and Z (“zigzag” or alternating measurements). Kaplan-Meier survival analysis revealed that pattern DI was significantly related to a higher death rate (*P* < 0.001) (Figure 8).

## Discussion

Great changes have taken place in traditional medicine after entry into the era of data. Physiological parameters can be recorded by wearable smart products (such as smart glasses, watches, and bracelets (49, 50), biological parameters can be expressed by gene sequencing (51), and anatomical parameters can be displayed by image data (52). The limits on analysis of such data by humans alone have clearly been exceeded, necessitating an increased reliance on machines. Accordingly, at the same time that there is more dependence than ever on humans to provide healthcare, algorithms are desperately needed to help (53).

Uveal melanoma (UM) is the most common intraocular tumor in adults. Although several treatments are available for patients with UM, more than half of patients end up with distant metastases. Unfortunately, there is currently no effective treatment for the metastatic disease, and the median survival time for metastatic UM is only 12 months (54–56). So risk factors that allow the early prediction of the metastasis and survival time of patients will contribute to the implementation of a more aggressive treatment strategy and improve patient outcomes (57). Additionally, numerous studies have shown that the great majority of patients want to know whether their prognosis is good or bad both before surgery and during follow-up. Although the bad news is particularly upsetting, patients feel a sense of empowerment over their future planning and a reduction in uncertainty and accompanying anxiety (58–60).

Our previous studies, and those of others, have shown that clinical characteristics such as male gender, advanced age, larger tumor size, epithelioid cell type, subretinal fluid, and ciliary body involvement can increase the risk of metastasis and death (10, 11, 61–64). Additionally, the treatment response by tumors can also affect the outcome to some extent. Several studies discovered that local treatment failure, defined by COMS as a 15% increase in tumor thickness after brachytherapy was significantly related to uveal melanoma-related mortality and systemic dissemination (15, 65). Furthermore, Augsburger and Kaiserman (19, 66) found that rapid regression of tumors after plaque brachytherapy indicates an unfavorable prognosis. Also, in other treatment modalities, Christoph et al. (67) reported a non-linear influence of the regression rate of choroidal melanoma as an independent risk factor of metastatic disease after linear accelerator stereotactic fractionated photon radiotherapy. Thus, tumor size change after surgery is significantly correlated with prognosis. In our research, we added this aspect to the construction of the model to determine whether postoperative information could improve model performance for prediction.

Medicine has experienced the recent emergence of artificial intelligence (AI) as a novel tool for analyzing large amounts of data (68). AI has recently achieved high accuracy in recognizing ocular structure. Deep-learning convolutinal neural networks (CNNs) developed by Li Dong et al. (69–71) have shown superior performance in assessing axial length, subfoveal choroidal thickness, and fundus tessellated density with color fundus photographs. In the diagnosis of multiple ocular disorders, AI outperformed human experts with multimodality imaging, including magnetic resonance imaging (MRI), fundus photographs, and fundus fluorescence angiography (FFA). An updated meta-analysis demonstrated that AI-based algorithms are capable of detecting age-related macular degeneration (AMD) in fundus images with a pooled AUC 0.983 (72, 73). Naoya Nezu et al. (74) recently reported that several algorithms predicted the diagnosis of 17 selected intraocular diseases including UM with aqueous humor cytokines, and indicated some new biomarkers facilitating the diagnosis of relevant diseases. In addition, Zhang et al. (75) also justified the effectiveness of deep learning for predicting nBAP1 expression in UM based only on Hematoxylin and eosin (H&E) sections.

Models based on clinical and demographic characteristics are being used to predict the prognosis of individual patients with UM after treatment. Jorge Vaquero-Garcia et al. (24)created an interactive web-based tool for the Prediction of Risk of Metastasis in Uveal Melanoma (PRiMeUM), which provides a tool for assessing the personalized risk for metastasis based on individual and tumor characteristics. The accuracy of the risk prediction was 80% using only chromosomal features, 83% using only clinical features, and 85% using combined clinical and chromosomal information. However, in most eye centers, chromosomal information is not available. Fine-needle aspiration biopsy is an invasive method and may contribute to some related complications such as vision loss, persistent hemorrhage, and even extraocular extension (76). Therefore, most patients being treated by plaque brachytherapy are reluctant to accept this examination.

We previously applied machine learning technology to establish a model to predict whether a patient would die or metastasize within 2 years after initial treatment. This model achieved an overall accuracy of 77.0 and 75.0% with all features (77). Information extracted from B-ultrasound images was additionally applied to machine learning to provide personalized risk prediction. To the best of our knowledge, ours is the first machine learning-based UM prognosis model using follow-up information after surgery. With the increasing availability of follow-up information, the performance of predictive models has improved significantly. The AUC of models increased from 0.708 to 0.883 after two additional follow-up records were added.

Figure 5 shows that follow-up data were remarkably correlated with 4-year survival. This suggests we can provide a more accurate prognostic evaluation for patients by intensive follow-up, which is readily obtained. In our study, tumor treatment response was divided into four patterns. The D pattern of decreasing tumor thickness correlated to the best prognosis, contrary to some previous research (18, 19). It found that early rapid regression of tumors after plaque brachytherapy was associated with an unfavorable outcome for patients with UM. However, a greater regression indicated a better prognosis in our relatively longer postoperative follow-up. In addition, similar to their results, a positive correlation between tumor thickness and regression rate was also found in our research.

Among the patients enrolled for model construction, the 177 surviving patients with UM with follow-up ranging from 3 to 4 years, can validate algorithms in a short time. Additionally, we welcome external datasets, especially with Asian patients, to continue our validation efforts. We hope that a predictive model for Asian patients can be established using factors that are non-invasive and easily available clinically in the future.

Deep learning (DL)-powered ultrasound has begun to be widely used in diagnosing certain diseases and for distinguishing between benign and malignant tumor types (78–80). But it has been used less for determining prognosis. Thus, we have also tried to construct a DL model using B-ultrasound images to predict long-term survival in patients with UM. However, the performance was found to be unsatisfactory. We do plan to undertake additional prospective studies that will incorporate uniform standard ultrasound images and color Doppler flow imaging to gather more prognostic information. In addition, multiple imaging modalities have been used recently with deep learning, including CT and MRI. Using these tools, researchers can attain more specific and informative histology and prognostic information. Compared to ultrasound, MRI provides excellent contrast resolution and multiple tissue-contrasts. Due to the paramagnetic effect, lesions with different melanin contents will present distinct signal intensities in MRI. Furthermore, the use of multiple sequences including dynamic contrast-enhanced (DCE) sequence and diffusion-weighted MR imaging has made it easier to identify intertumor heterogeneity (81, 82). It has been proven that quantitative multiparametric MRI can be used to predict monosomy 3 and UM metastasis (83, 84). Therefore, we propose to adopt DL to automatically extract high-throughput features from multi-modal, multi-channel preoperative MRI to predict the survival time for patients with UM. This will enable us to better develop personalized treatment plans and realize precision medicine.

There are some limitations in our study that should be noted. First, while death is an outcome that can be precisely determined metastasis can only be detected at follow-up visits. Therefore, metastasis may present before the clinical diagnosis, which would affect our model's predictive value for metastasis. Second, due to the retrospective nature of this study, the follow-up interval after surgery in our study was not fixed. This affected the results to some extent. Third, based on the COMS data, post-therapy surveillance relies on decreasing thickness with ultrasound B repeated every 6 months for 2 years and yearly after that (16). But most of our patients can only be checked three times within 3 years. Our results showed that the algorithm's performance could be enhanced with more follow-up visits. Frequent follow-up of patients is advisable, ideally leading to earlier detection of metastasis and timely enrollment into treatment and care. Thus, patients will be strictly followed up in the future to further explore the role of data from follow-up examinations in predicting prognosis.

## Conclusions

In conclusion, the present study developed an RF model to predict the risk of UM metastasis and death within 4 years based on ultrasound follow-up records following plaque brachytherapy. We intend to further validate our model in prospective datasets, which can prompt us to implement timely and efficient treatments.

## Data Availability Statement

The raw data supporting the conclusions of this article will be made available by the authors, without undue reservation.

## Ethics Statement

The studies involving human participants were reviewed and approved by Beijing Tongren Hospital of Capital Medical University. The patients/participants provided their written informed consent to participate in this study.

## Author Contributions

YaL and WW contributed to the concept, design of the study, revised the manuscript, and handled the supervision. JL, YC, and YY wrote the manuscript. JL developed the study. YC, YY, KZ, YuL, HZ, LD, and JX participated in the final design of the study. JL, YC, YY, KZ, YuL, and HZ carried out the study. JL, YY, YuL, and HZ collected the data. All authors read and approved the final submitted version of the manuscript.

## Funding

This trial is supported by the National Natural Science Foundation of China (82101180); Beijing Natural Science Foundation (7204245); Scientific Research Common Program of Beijing Municipal Commission of Education (KM202010025018); Beijing Municipal Administration of Hospital's Youth Programme (QML20190202); Beijing Dongcheng District Outstanding Talents Cultivating Plan (2018); the Capital Health Research and Development of Special (2020-1-2052); Science & Technology Project of Beijing Municipal Science & Technology Commission (Z201100005520045, Z181100001818003).

## Conflict of Interest

KZ is employed by InferVision Healthcare Science and Technology Limited Company. The remaining authors declare that the research was conducted in the absence of any commercial or financial relationships that could be construed as a potential conflict of interest.

## Publisher's Note

All claims expressed in this article are solely those of the authors and do not necessarily represent those of their affiliated organizations, or those of the publisher, the editors and the reviewers. Any product that may be evaluated in this article, or claim that may be made by its manufacturer, is not guaranteed or endorsed by the publisher.

## References

[1] Hu DN Yu GP McCormick SA Schneider S Finger PT. Population-based incidence of uveal melanoma in various races and ethnic groups. Am J Ophthalmol. (2005) 140:612–7. 10.1016/j.ajo.2005.05.034 16226513

[2] Park SJ Oh CM Kim BW Woo SJ Cho H Park KH. Nationwide incidence of ocular melanoma in South Korea by using the national cancer registry database (1999-2011). Invest Ophthalmol Vis Sci. (2015) 56:4719–24. 10.1167/iovs.15-16532 26207308

[3] Stang A Parkin DM Ferlay J Jöckel KH. International uveal melanoma incidence trends in view of a decreasing proportion of morphological verification. Int J Cancer. (2005) 114:114–23. 10.1002/ijc.20690 15523698

[4] Tomizuka T Namikawa K Higashi T. Characteristics of melanoma in Japan: a nationwide registry analysis 2011-2013. Melanoma Res. (2017) 27:492–7. 10.1097/CMR.0000000000000375 28609317

[5] Sikuade MJ Salvi S Rundle PA Errington DG Kacperek A Rennie IG. Outcomes of treatment with stereotactic radiosurgery or proton beam therapy for choroidal melanoma. Eye. (2015) 29:1194–8. 10.1038/eye.2015.109 26160531 PMC4565940

[6] Rajeshuni N Zubair T Ludwig CA Moshfeghi DM Mruthyunjaya P. Evaluation of racial, ethnic, and socioeconomic associations with treatment and survival in uveal melanoma, 2004-2014. JAMA Ophthalmol. (2020) 138:876–84. 10.1001/jamaophthalmol.2020.2254 32614376 PMC7333178

[7] Singh AD Turell ME Topham AK. Uveal melanoma: trends in incidence, treatment, and survival. Ophthalmology. (2011) 118:1881–5. 10.1016/j.ophtha.2011.01.040 21704381

[8] Kujala E Mäkitie T Kivelä T. Very long-term prognosis of patients with malignant uveal melanoma. Invest Ophthalmol Vis Sci. (2003) 44:4651–9. 10.1167/iovs.03-0538 14578381

[9] Diener-West M Reynolds SM Agugliaro DJ Caldwell R Cumming K Earle JD. Development of metastatic disease after enrollment in the COMS trials for treatment of choroidal melanoma: collaborative ocular melanoma study group report no. 26. Arch Ophthalmol. (2005) 123:1639–43. 10.1001/archopht.123.12.1639 16344433

[10] Delgado-Ramos GM Thomas F VanderWalde A King B Wilson M Pallera AM. Risk factors, clinical outcomes, and natural history of uveal melanoma: a single-institution analysis. Med Oncol. (2019) 36:17. 10.1007/s12032-018-1230-4 30666496

[11] Shields CL Kaliki S Cohen MN Shields PW Furuta M Shields JA. Prognosis of uveal melanoma based on race in 8100 patients: the 2015 doyne lecture. Eye. (2015) 29:1027–35. 10.1038/eye.2015.51 26248525 PMC4541345

[12] Broggi G Russo A Reibaldi M Russo D Varricchio S Bonfiglio V. Histopathology and genetic biomarkers of choroidal melanoma. Appl Sci. (2020) 10:8081. 10.3390/app10228081

[13] Jager MJ Shields CL Cebulla CM Abdel-Rahman MH Grossniklaus HE Stern MH. Uveal melanoma. Nat Rev Dis Primers. (2020) 6:24. 10.1038/s41572-020-0158-0 32273508

[14] Chang MY McCannel TA. Local treatment failure after globe-conserving therapy for choroidal melanoma. Br J Ophthalmol. (2013) 97:804–11. 10.1136/bjophthalmol-2012-302490 23645818 PMC3686322

[15] Ophthalmic Oncology Task Force. Local recurrence significantly increases the risk of metastatic uveal melanoma. Ophthalmology. (2016) 123:86–91. 10.1016/j.ophtha.2015.09.014 26505803

[16] Stålhammar G. Forty-year prognosis after plaque brachytherapy of uveal melanoma. Sci Rep. (2020) 10:11297. 10.1038/s41598-020-68232-7 32647177 PMC7347921

[17] Jampol LM Moy CS Murray TG Reynolds SM Albert DM Schachat AP. The COMS randomized trial of iodine 125 brachytherapy for choroidal melanoma: IV. local treatment failure and enucleation in the first 5 years after brachytherapy COMS report no 19. Ophthalmology. (2002) 109:2197–206. 10.1016/S0161-6420(02)01277-0 12466159

[18] Augsburger JJ Gamel JW Shields JA Markoe AM Brady LW. Post-irradiation regression of choroidal melanomas as a risk factor for death from metastatic disease. Ophthalmology. (1987) 94:1173–7. 10.1016/S0161-6420(87)33310-X 3684235

[19] Kaiserman I Anteby I Chowers I Blumenthal EZ Kliers I Pe'er J. Post-brachytherapy initial tumour regression rate correlates with metastatic spread in posterior uveal melanoma. Br J Ophthalmol. (2004) 88:892–5. 10.1136/bjo.2003.036285 15205232 PMC1772205

[20] Damato B Eleuteri A Taktak AF Coupland SE. Estimating prognosis for survival after treatment of choroidal melanoma. Prog Retin Eye Res. (2011) 30:285–95. 10.1016/j.preteyeres.2011.05.003 21658465

[21] Eleuteri A Damato B Coupland S Taktak A. Enhancing survival prognostication in patients with choroidal melanoma by integrating pathologic, clinical and genetic predictors of metastasis. Int J Biomed Eng Technol. (2012) 8:18–35. 10.1504/IJBET.2012.04535535009967

[22] Cunha Rola A Taktak A Eleuteri A Kalirai H Heimann H Hussain R. Multicenter external validation of the liverpool uveal melanoma prognosticator online: an OOG collaborative study. Cancers. (2020) 12:477. 10.3390/cancers12020477 32085617 PMC7072188

[23] DeParis SW Taktak A Eleuteri A Enanoria W Heimann H Coupland SE. External validation of the liverpool uveal melanoma prognosticator online. Invest Ophthalmol Vis Sci. (2016) 57:6116–22. 10.1167/iovs.16-19654 27835710

[24] Vaquero-Garcia J Lalonde E Ewens KG Ebrahimzadeh J Richard-Yutz J Shields CL. PRiMeUM: a model for predicting risk of metastasis in uveal melanoma. Invest Ophthalmol Vis Sci. (2017) 58:4096–105. 10.1167/iovs.17-22255 28828481 PMC6108308

[25] Damato B Eleuteri A Hussain R Kalirai H Thornton S Taktak A. Parsimonious models for predicting mortality from choroidal melanoma. Invest Ophthalmol Vis Sci. (2020) 61:35. 10.1167/iovs.61.4.35 32334433 PMC7401884

[26] Eleuteri A Taktak AFG Coupland SE Heimann H Kalirai H Damato B. Prognostication of metastatic death in uveal melanoma patients: a markov multi-state model. Comput Biol Med. (2018) 102:151–6. 10.1016/j.compbiomed.2018.09.024 30278339

[27] Lin D Chen J Lin Z Li X Zhang K Wu X. A practical model for the identification of congenital cataracts using machine learning. EBioMedicine. (2020) 51:102621. 10.1016/j.ebiom.2019.102621 31901869 PMC6948173

[28] Li W Yang Y Zhang K Long E He L Zhang L. Dense anatomical annotation of slit-lamp images improves the performance of deep learning for the diagnosis of ophthalmic disorders. Nat Biomed Eng. (2020) 4:767–77. 10.1038/s41551-020-0577-y 32572198

[29] Hannun AY Rajpurkar P Haghpanahi M Tison GH Bourn C Turakhia MP. Cardiologist-level arrhythmia detection and classification in ambulatory electrocardiograms using a deep neural network. Nat Med. (2019) 25:65–9. 10.1038/s41591-018-0268-3 30617320 PMC6784839

[30] Yang J Zhang K Fan H Huang Z Xiang Y Yang J. Development and validation of deep learning algorithms for scoliosis screening using back images. Commun Biol. (2019) 2:390. 10.1038/s42003-019-0635-8 31667364 PMC6814825

[31] Zhang Y Li F Yuan F Zhang K Huo L Dong Z. Diagnosing chronic atrophic gastritis by gastroscopy using artificial intelligence. Dig Liver Dis. (2020) 52:566–72. 10.1016/j.dld.2019.12.146 32061504

[32] Wang L Zhang K Liu X Long E Jiang J An Y. Comparative analysis of image classification methods for automatic diagnosis of ophthalmic images. Sci Rep. (2017) 7:41545. 10.1038/srep41545 28139688 PMC5282520

[33] Mucaki EJ Zhao JZL Lizotte DJ Rogan PK. Predicting responses to platin chemotherapy agents with biochemically-inspired machine learning. Signal Transduct Target Ther. (2019) 4:1. 10.1038/s41392-018-0034-5 30652029 PMC6329797

[34] Fleuren LM Klausch TLT Zwager CL Schoonmade LJ Guo T Roggeveen LF. Machine learning for the prediction of sepsis: a systematic review and meta-analysis of diagnostic test accuracy. Intensive Care Med. (2020) 46:383–400. 10.1007/s00134-019-05872-y 31965266 PMC7067741

[35] Park SM Won DD Lee BJ Escobedo D Esteva A Aalipour A. A mountable toilet system for personalized health monitoring via the analysis of excreta. Nat Biomed Eng. (2020) 4:624–35. 10.1038/s41551-020-0534-9 32251391 PMC7377213

[36] Liu Y Jain A Eng C Way DH Lee K Bui P. A deep learning system for differential diagnosis of skin diseases. Nat Med. (2020) 26:900–8. 10.1038/s41591-020-0842-3 32424212

[37] Mei X Lee HC Diao KY Huang M Lin B Liu C. Artificial intelligence-enabled rapid diagnosis of patients with COVID-19. Nat Med. (2020) 26:1224–8. 10.1038/s41591-020-0931-3 32427924 PMC7446729

[38] Kather JN Pearson AT Halama N Jäger D Krause J Loosen SH. Deep learning can predict microsatellite instability directly from histology in gastrointestinal cancer. Nat Med. (2019) 25:1054–6. 10.1038/s41591-019-0462-y 31160815 PMC7423299

[39] Breiman L. Random forests. Mach Learn. (2001) 45:5–32. 10.1023/A:1010933404324

[40] Zhang X Zhang K Lin D Zhu Y Chen C He L. Artificial intelligence deciphers codes for color and odor perceptions based on large-scale chemoinformatic data. Gigascience. (2020) 9:giaa011. 10.1093/gigascience/giaa011 32101298 PMC7043059

[41] Zhang K Liu X Jiang J Li W Wang S Liu L. Prediction of postoperative complications of pediatric cataract patients using data mining. J Transl Med. (2019) 17:2. 10.1186/s12967-018-1758-2 30602368 PMC6317183

[42] Hyland SL Faltys M Hüser M Lyu X Gumbsch T Esteban C. Early prediction of circulatory failure in the intensive care unit using machine learning. Nat Med. (2020) 26:364–73. 10.1038/s41591-020-0789-4 32152583

[43] Sim H Lee J. Cost-effective stochastic MAC circuits for deep neural networks. Neural Netw. (2019) 117:152–62. 10.1016/j.neunet.2019.04.017 31170575

[44] Liu XY Wu J Zhou ZH. Exploratory undersampling for class-imbalance learning. IEEE Trans Syst Man Cybern B Cybern. (2009) 39:539–50. 10.1109/TSMCB.2008.2007853 19095540

[45] Zhang K Liu X Liu F He L Zhang L Yang Y. An interpretable and expandable deep learning diagnostic system for multiple ocular diseases: qualitative study. J Med Internet Res. (2018) 20:e11144. 10.2196/11144 30429111 PMC6301833

[46] Zhang K Li X He L Guo C Yang Y Dong Z. A human-in-the-loop deep learning paradigm for synergic visual evaluation in children. Neural Netw. (2020) 122:163–73. 10.1016/j.neunet.2019.10.003 31683144

[47] Fang R Wang H Li Y Liu YM Wei WB. Regression patterns of uveal melanoma after iodine-125 plaque brachytherapy. BMC Ophthalmol. (2021) 21:137. 10.1186/s12886-021-01898-3 33726696 PMC7968312

[48] Rashid M Heikkonen J Kivelä T. Tumor regression after brachytherapy for choroidal melanoma: reduction of thickness and cross-sectional area by shape and regression pattern. Invest Ophthalmol Vis Sci. (2015) 56:2612–23. 10.1167/iovs.14-16322 25813993

[49] Voelker R. Smart watch detects seizures. JAMA. (2018) 319:1086. 10.1001/jama.2018.1809 29558540

[50] Ahn D Chung H Lee HW Kang K Ko PW Kim NS. Smart gait-aid glasses for parkinson's disease patients. IEEE Trans Biomed Eng. (2017) 64:2394–402. 10.1109/TBME.2017.2655344 28113199

[51] Libbrecht MW Noble WS. Machine learning applications in genetics and genomics. Nat Rev Genet. (2015) 16:321–32. 10.1038/nrg3920 25948244 PMC5204302

[52] Ehteshami Bejnordi B Veta M Johannes van Diest P van Ginneken B Karssemeijer N Litjens G. Diagnostic assessment of deep learning algorithms for detection of lymph node metastases in women with breast cancer. JAMA. (2017) 318:2199–210. 10.1001/jama.2017.14585 29234806 PMC5820737

[53] Topol EJ. High-performance medicine: the convergence of human and artificial intelligence. Nat Med. (2019) 25:44–56. 10.1038/s41591-018-0300-7 30617339

[54] Komatsubara KM Carvajal RD. Immunotherapy for the treatment of uveal melanoma: current status and emerging therapies. Curr Oncol Rep. (2017) 19:45. 10.1007/s11912-017-0606-5 28508938

[55] Collaborative Ocular Melanoma Study Group. Assessment of metastatic disease status at death in 435 patients with large choroidal melanoma in the collaborative ocular melanoma study (COMS): COMS report no. 15. Arch Ophthalmol. (2001) 119:670–6. 10.1001/archopht.119.5.670 11346394

[56] Khoja L Atenafu EG Suciu S Leyvraz S Sato T Marshall E. Meta-analysis in metastatic uveal melanoma to determine progression free and overall survival benchmarks: an international rare cancers initiative (IRCI) ocular melanoma study. Ann Oncol. (2019) 30:1370–80. 10.1093/annonc/mdz176 31150059

[57] Damato B. Ocular treatment of choroidal melanoma in relation to the prevention of metastatic death - a personal view. Prog Retin Eye Res. (2018) 66:187–99. 10.1016/j.preteyeres.2018.03.004 29571968

[58] Williamson TJ Jorge-Miller A McCannel TA Beran TM Stanton AL. Sociodemographic, medical, and psychosocial factors associated with supportive care needs in adults diagnosed with uveal melanoma. JAMA Ophthalmol. (2018) 136:356–63. 10.1001/jamaophthalmol.2018.0019 29470565 PMC5876854

[59] Erim Y Scheel J Breidenstein A Metz CH Lohmann D Friederich HC. Psychosocial impact of prognostic genetic testing in the care of uveal melanoma patients: protocol of a controlled prospective clinical observational study. BMC Cancer. (2016) 16:408. 10.1186/s12885-016-2479-7 27386847 PMC4936050

[60] Cook SA Damato B Marshall E Salmon P. Psychological aspects of cytogenetic testing of uveal melanoma: preliminary findings and directions for future research. Eye. (2009) 23:581–5. 10.1038/eye.2008.54 18344957

[61] Zloto O Pe'er J Frenkel S. Gender differences in clinical presentation and prognosis of uveal melanoma. Invest Ophthalmol Vis Sci. (2013) 54:652–6. 10.1167/iovs.12-10365 23197684

[62] Liu YM Li Y Wei WB Xu X Jonas JB. Clinical characteristics of 582 patients with uveal melanoma in China. PLoS ONE. (2015) 10:e0144562. 10.1371/journal.pone.0144562 26645696 PMC4672905

[63] Shields CL Furuta M Thangappan A Nagori S Mashayekhi A Lally DR. Metastasis of uveal melanoma millimeter-by-millimeter in 8033 consecutive eyes. Arch Ophthalmol. (2009) 127:989–98. 10.1001/archophthalmol.2009.208 19667335

[64] Rietschel P Panageas KS Hanlon C Patel A Abramson DH Chapman PB. Variates of survival in metastatic uveal melanoma. J Clin Oncol. (2005) 23:8076–80. 10.1200/JCO.2005.02.6534 16258106

[65] Vrabec TR Augsburger JJ Gamel JW Brady LW Hernandez C Woodleigh R. Impact of local tumor relapse on patient survival after cobalt 60 plaque radiotherapy. Ophthalmology. (1991) 98:984–8. 10.1016/S0161-6420(91)32193-6 1866154

[66] Cruess AF Augsburger JJ Shields JA Brady LW Markoe AM Day JL. Regression of posterior uveal melanomas following cobalt-60 plaque radiotherapy. Ophthalmology. (1984) 91:1716–9. 10.1016/S0161-6420(84)34087-8 6522001

[67] Mitsch C Zehetmayer M Gleiss A Georg D Dieckmann K Pötter R. Early ultrasonographic tumor regression after linear accelerator stereotactic fractionated photon radiotherapy of choroidal melanoma as a predictor for metastatic spread. Radiother Oncol. (2018) 127:385–91. 10.1016/j.radonc.2018.04.019 29747872

[68] Yu KH Beam AL Kohane IS. Artificial intelligence in healthcare. Nat Biomed Eng. (2018) 2:719–31. 10.1038/s41551-018-0305-z 31015651

[69] Dong L Hu XY Yan YN Zhang Q Zhou N Shao L. Deep learning-based estimation of axial length and subfoveal choroidal thickness from color fundus photographs. Front Cell Dev Biol. (2021) 9:653692. 10.3389/fcell.2021.653692 33898450 PMC8063031

[70] Shao L Zhang QL Long TF Dong L Zhang C Da Zhou W. Quantitative assessment of fundus tessellated density and associated factors in fundus images using artificial intelligence. Transl Vis Sci Technol. (2021) 10:23. 10.1167/tvst.10.9.23 34406340 PMC8383900

[71] Cheung CY Xu D Cheng CY Sabanayagam C Tham YC Yu M. A deep-learning system for the assessment of cardiovascular disease risk via the measurement of retinal-vessel calibre. Nat Biomed Eng. (2021) 5:498–508. 10.1038/s41551-020-00626-4 33046867

[72] Dong L Yang Q Zhang RH Wei WB. Artificial intelligence for the detection of age-related macular degeneration in color fundus photographs: a systematic review and meta-analysis. EClinicalMedicine. (2021) 35:100875. 10.1016/j.eclinm.2021.100875 34027334 PMC8129891

[73] Lin D Xiong J Liu C Zhao L Li Z Yu S. Application of comprehensive artificial intelligence retinal expert (CARE) system: a national real-world evidence study. Lancet Digit Health. (2021) 3:e486–e95. 10.1016/S2589-7500(21)00086-8 34325853

[74] Nezu N Usui Y Saito A Shimizu H Asakage M Yamakawa N. Machine learning approach for intraocular disease prediction based on aqueous humor immune mediator profiles. Ophthalmology. (2021) 128:1197–208. 10.1016/j.ophtha.2021.01.019 33484732

[75] Zhang H Kalirai H Acha-Sagredo A Yang X Zheng Y Coupland SE. Piloting a deep learning model for predicting nuclear BAP1 immunohistochemical expression of uveal melanoma from hematoxylin-and-eosin sections. Transl Vis Sci Technol. (2020) 9:50. 10.1167/tvst.9.2.50 32953248 PMC7476670

[76] Singh AD Medina CA Singh N Aronow ME Biscotti CV Triozzi PL. Fine-needle aspiration biopsy of uveal melanoma: outcomes and complications. Br J Ophthalmol. (2016) 100:456–62. 10.1136/bjophthalmol-2015-306921 26231747

[77] Chen YN Wang YN Chen MX Zhang K Chen RT Fang R. Machine learning models for outcome prediction of Chinese uveal melanoma patients: A 15-year follow-up study. Cancer Commun. (2022). 10.1002/cac2.12253 35001563 PMC8923127

[78] Liang X Yu J Liao J Chen Z. Convolutional neural network for breast and thyroid nodules diagnosis in ultrasound imaging. Biomed Res Int. (2020) 2020:1763803. 10.1155/2020/1763803 32420322 PMC7199615

[79] Brattain LJ Telfer BA Dhyani M Grajo JR Samir AE. Machine learning for medical ultrasound: status, methods, and future opportunities. Abdom Radiol. (2018) 43:786–99. 10.1007/s00261-018-1517-0 29492605 PMC5886811

[80] Chen J You H Li K. A review of thyroid gland segmentation and thyroid nodule segmentation methods for medical ultrasound images. Comput Methods Programs Biomed. (2020) 185:105329. 10.1016/j.cmpb.2020.105329 31955006

[81] Foti PV Travali M Farina R Palmucci S Spatola C Raffaele L. Diagnostic methods and therapeutic options of uveal melanoma with emphasis on MR imaging-part I: MR imaging with pathologic correlation and technical considerations. Insights Imaging. (2021) 12:66. 10.1186/s13244-021-01000-x 34080069 PMC8172816

[82] Foti PV Travali M Farina R Palmucci S Spatola C Liardo RLE. Diagnostic methods and therapeutic options of uveal melanoma with emphasis on MR imaging-Part II: treatment indications and complications. Insights Imaging. (2021) 12:67. 10.1186/s13244-021-01001-w 34085131 PMC8175681

[83] Kamrava M Sepahdari AR Leu K Wang PC Roberts K Demanes DJ. Quantitative multiparametric MRI in uveal melanoma: increased tumor permeability may predict monosomy 3. Neuroradiology. (2015) 57:833–40. 10.1007/s00234-015-1546-0 26022354

[84] Wei W Jia G von Tengg-Kobligk H Heverhagen JT Abdel-Rahman M Wei L. Dynamic contrast-enhanced magnetic resonance imaging of ocular melanoma as a tool to predict metastatic potential. J Comput Assist Tomogr. (2017) 41:823–7. 10.1097/RCT.0000000000000598 28448404

