# Supplementary material for: Prognosis Prediction of Uveal Melanoma After Plaque Brachytherapy Based on Ultrasound With Machine Learning
Source: Front Med (Lausanne). 2022 Jan 21;8:777142. doi: 10.3389/fmed.2021.777142 (PMC8816318; doi:10.3389/fmed.2021.777142)
Supplement: Supplementary file 1 [file Table_1.DOCX]

**Supplementary Table 1|**Baseline characteristics of the survival and death cohorts (left), metastasis and non-metastasis cohorts (right).

| Characteristics | Survival(N=402) | Death(N=52) | *P* | Metastasis(N=48) | Non-metastasis(N=376) | *P* |
| --- | --- | --- | --- | --- | --- | --- |
| Age, years | 46.15±11.58 | 47.27±12.60 | 0.518 | 46.38±10.67 | 46.21±11.63 | 0.972 |
| Corrected visual acuity | 0.35±0.35 | 0.32±0.30 | 0.488 | 0.34±0.33 | 0.35±0.34 | 0.840 |
| Intraocular pressure, mm | 13.29±2.91 | 14.30±5.49 | 0.198 | 13.42±4.68 | 13.41±2.94 | 0.924 |
| Maximum basal diameter, mm | 11.81±2.91 | 13.13±2.99 | 0.002 | 13.77±3.16 | 11.70±2.81 | ＜0.001 |
| Minimum basal diameter, mm | 10.42±2.68 | 11.35±2.37 | 0.018 | 12.15±2.52 | 10.29±2.62 | ＜0.001 |
| Maximal thickness, mm | 6.67±2.36 | 7.03±2.69 | 0.321 | 7.12±2.27 | 6.67±2.38 | 0.213 |
| Time to the first follow-up, days | 148 [120,222] | 241 [164,319] | ＜0.001 | 167 [127,318] | 156[127,318] | 0.171 |
| Maximum basal diameter at the first follow-up, mm | 11.35±2.57 | 12.82±2.60 | ＜0.001 | 13.36±2.67 | 11.21±2.45 | ＜0.001 |
| Minimum basal diameter at the first follow-up, mm | 10.24±2.39 | 11.31±2.11 | 0.002 | 11.90±2.26 | 10.09±2.25 | ＜0.001 |
| Maximum thickness at the first follow-up, mm | 6.14±2.08 | 6.24±2.11 | 0.737 | 6.65±2.00 | 6.08±2.08 | 0.074 |
| Time to the second follow-up, days | 406 [310,549] | 382 [335,427] | 0.333 | 390 [310,482] | 448 [314,612] | 0.032 |
| Maximum basal diameter at the second follow-up, mm | 11.12±2.53 | 12.99±2.21 | ＜0.001 | 13.17±2.42 | 10.97±2.38 | ＜0.001 |
| Minimum basal diameter at the second follow-up, mm | 10.02±2.27 | 11.00±1.91 | ＜0.001 | 11.09±2.08 | 9.92±2.14 | 0.001 |
| Maximum thickness at the second follow-up, mm | 5.89±2.22 | 6.74±1.80 | 0.008 | 6.47±1.98 | 5.85±2.20 | 0.063 |
| Time to the third follow-up, days | 709 [574,860] | 641 [603,686] | ＜0.001 | 674 [560,716] | 746 [574,919] | ＜0.001 |
| Maximum basal diameter at the third follow-up, mm | 11.07±2.62 | 13.80±2.09 | ＜0.001 | 13.67±2.67 | 10.85±2.47 | ＜0.001 |
| Minimum basal diameter at the third follow-up, mm | 10.02±2.36 | 11.96±1.96 | ＜0.001 | 12.14±2.14 | 9.82±2.21 | ＜0.001 |
| Maximum thickness at the third follow-up, mm | 5.68±2.04 | 8.25±1.86 | ＜0.001 | 7.20±2.15 | 5.63±2.07 | ＜0.001 |
| T specific stage, n (%) |  |  | 0.040 |  |  | 0.033 |
| 1a | 37(9.2%) | 4(7.7%) |  | 3(6.3%) | 33(8.8%) |  |
| 1b | 4(1.0%) | 0 |  | 15(31.3%) | 4(1.1%) |  |
| 2a | 209(52.0%) | 17(32.7%) |  | 1(2.1%) | 201(53.5%) |  |
| 2b | 4(1.0%) | 1(1.9%) |  | 20(41.7%) | 4(1.1%) |  |
| 3a | 118(29.4%) | 20(38.5%) |  | 7(14.6%) | 107(28.5%) |  |
| 3b | 19(4.7%) | 8(15.4%) |  | 1(2.1%) | 18(4.8%) |  |
| 4a | 5(1.2%) | 1(1.9%) |  | 1(2.1%) | 4(1.1%) |  |
| 4b | 6(1.5%) | 1(1.9%) |  | 47(11.4%) | 5(1.3%) |  |
| Ciliary body involvement, n (%) |  |  | 0.021 |  |  | 0.037 |
| yes | 33(8.2%) | 10(19.2%) |  | 39(81.3%) | 345(91.8%) |  |
| no | 369(91.8%) | 42(80.8%) |  | 9(18.8%) | 31(8.2%) |  |
| Subretinal fluid, n (%) |  |  | 0.040 |  |  | 0.001 |
| yes | 286(71.1%) | 44(84.6%) |  | 4(8.3%) | 115(30.6%) |  |
| no | 116(28.9%) | 8(15.4%) |  | 44(91.7%) | 261(69.4%) |  |
| Optic disk involvement, n (%) |  |  | 1.000 |  |  | 1.000 |
| yes | 9(2.2%) | 1(1.9%) |  | 47(97.9%) | 367(97.6%) |  |
| no | 393(97.8%) | 51(98.1%) |  | 1(2.1%) | 9(2.4%) |  |
| Vitreous hemorrhage, n (%) |  |  | 1.000 |  |  | 0.234 |
| yes | 25(6.2%) | 3(5.8%) |  | 47(97.9%) | 352(93.6%) |  |
| no | 377(93.8%) | 49(94.2%) |  | 1(2.1%) | 24(6.4%) |  |
| Laterality, n (%) |  |  | 0.905 |  |  | 0.767 |
| right | 220(54.7%) | 28(53.8%) |  | 27(56.3%) | 203(54.0%) |  |
| left | 182(45.3%) | 24(46.2%) |  | 21(43.8%) | 173(46.0%) |  |
| Origin, n (%) |  |  | 0.174 |  |  | 0.609 |
| iris | 0 | 0 |  | 0 | 0 |  |
| ciliary body | 7(1.7%) | 3(5.8%) |  | 2(4.2%) | 7(1.9%) |  |
| choroid | 395(98.3%) | 49(94.2%) |  | 46(95.8%) | 369(98.1%) |  |
| Configuration, n (%) |  |  |  |  |  | 0.078 |
| mushroom | 121(30.1%) | 13(25%) |  | 11(22.9%) | 118(31.4%) |  |
| flat | 7(1.7%) | 0 |  | 1(2.1%) | 6(1.6%) |  |
| hemisphere | 247(61.4%) | 31(59.6%) |  | 28(58.3%) | 228(60.6%) |  |
| irregular | 25(6.2%) | 8(15.4%) |  | 7(14.6%) | 23(6.1%) |  |
| diffuse | 2(0.5%) | 0 |  | 1(2.1%) | 1(0.3%) |  |
| Position, n (%) |  |  | 0.037 |  |  | 0.089 |
| superior | 22(5.2%) | 7(13.7%) |  | 8(16.7%) | 19(5.1%) |  |
| nasal | 33(8.3%) | 4(7.4%) |  | 6(12.5%) | 26(6.9%) |  |
| inferior | 23(5.2%) | 2(3.2%) |  | 3(6.3%) | 23(6.1%) |  |
| temporal | 81(20.8%) | 11(21.1%) |  | 9(18.8%) | 78(20.7%) |  |
| superior temporal | 97(24.9%) | 7(13.7%) |  | 10(20.8%) | 93(24.7%) |  |
| superior nasal | 29(7.2%) | 5(9.5%) |  | 2(4.2%) | 24(6.4%) |  |
| inferior nasal | 37(9.3%) | 4(7.4%) |  | 2(4.2%) | 34(9.0%) |  |
| inferior temporal | 75(18.7%) | 8(15.8%) |  | 7(14.6%) | 74(19.7%) |  |
| macula | 3(0.3%) | 2(3.2%) |  | 0 | 3(0.8%) |  |
| bifocal | 2(0.2%) | 2(3.2%) |  | 1(2.1%) | 2(0.5%) |  |
| Gender, n (%) |  |  | 0.243 |  |  | 0.602 |
| male | 182(45.3%) | 28(53.8%) |  | 24(50.0%) | 173(46.0%) |  |
| female | 220(54.7%) | 24(46.2%) |  | 24(50.0%) | 203(54.0%) |  |
| Pattern, n (%) |  |  | ＜0.001 |  |  | 0.003 |
| D | 195(48.5%) | 13(25.0%) |  | 18(37.5%) | 190(50.5%) |  |
| S | 133(33.1%) | 6(11.5%) |  | 11(22.9%) | 121(32.2%) |  |
| I | 40(10.0%) | 15(28.8%) |  | 9(18.8%) | 37(9.8%) |  |
| O | 34(8.5%) | 18(34.6%) |  | 10(20.8%) | 28(7.4%) |  |

**Supplementary Table2 |**Models performance

|  | Cost sensitive | Accuracy | Sensitivity | Specificity | AUC |
| --- | --- | --- | --- | --- | --- |
|  | parameter | Mean (standard deviation) [95% confidence interval] | | | |
| Death within 4 years | 70 | 0.8302(0.0498) | 0.8045(0.0411) | 0.8335 (0.0517) | 0.8828(0.0259) |
| with three consecutive follow-ups |  | [0.7325 0.9278] | [0.7240 0.8850] | [0.7322 0.9347] | [0.8321 0.9335] |
| Death within 4 years | 70 | 0.5851(0.0738) | 0.7051(0.0769) | 0.5696(0.0831) | 0.7077(0.0452) |
| with one follow-up |  | [0.4405 0.7298] | [0.5544 0.8558] | [0.4068 0.7324] | [0.6192 0.7963] |
| Metastasis within 4 years | 50 | 0.7948(0.0312) | 0.7708(0.0798) | 0.7979(0.0378) | 0.8464(0.0257) |
| with three consecutive follow-ups |  | [0.7337 0.8559] | [0.6145 0.9271] | [0.7237 0.8721] | [0.7959 0.8968] |
| Metastasis within during 4 years | 50 | 0.6910(0.0356) | 0.6667(0.2041) | 0.6942 (0.0280) | 0.7297(0.0623) |
| with one follow-up |  | [0.6212 0.7608] | [0.2666 1.0000] | [0.6393 0.7490] | [0.6076 0.8518] |
